# Rehabilitation Technologies for Chronic Conditions: Will We Sink or Swim?

**DOI:** 10.3390/healthcare11202751

**Published:** 2023-10-17

**Authors:** Amber LaMarca, Ivy Tse, Julie Keysor

**Affiliations:** 1 Rehabilitation Sciences, MGH Institute of Health Professions, Boston, MA 02129, USA; alamarca@mghihp.edu; 2 Doctor of Physical Therapy Program, MGH Institute of Health Professions, Boston, MA 02129, USA; 3 School of Health Care Leadership, MGH Institute of Health Professions, Boston, MA 02129, USA

**Keywords:** rehabilitation technology, exoskeletons, virtual reality, augmentative reality, mHealth, remote monitoring

## Abstract

Introduction: Chronic conditions such as stroke, Parkinson’s disease, spinal cord injury, multiple sclerosis, vestibular disorders, chronic pain, arthritis, diabetes, chronic obstructive pulmonary disease (COPD), and heart disease are leading causes of disability among middle-aged and older adults. While evidence-based treatment can optimize clinical outcomes, few people with chronic conditions engage in the recommended levels of exercise for clinical improvement and successful management of their condition. Rehabilitation technologies that can augment therapeutic care—i.e., exoskeletons, virtual/augmented reality, and remote monitoring—offer the opportunity to bring evidence-based rehabilitation into homes. Successful integration of rehabilitation techniques at home could help recovery and access and foster long term self-management. However, widespread uptake of technology in rehabilitation is still limited, leaving many technologies developed but not adopted. Methods: In this narrative review, clinical need, efficacy, and obstacles and suggestions for implementation are discussed. The use of three technologies is reviewed in the management of the most prevalent chronic diseases that utilize rehabilitation services, including common neurological, musculoskeletal, metabolic, pulmonary, and cardiac conditions. The technologies are (i) exoskeletons, (ii) virtual and augmented reality, and (iii) remote monitoring. Results: Effectiveness evidence backing the use of technology in rehabilitation is growing but remains limited by high heterogeneity, lack of long-term outcomes, and lack of adoption outcomes. Conclusion: While rehabilitation technologies bring opportunities to bridge the gap between clinics and homes, there are many challenges with adoption. Hybrid effectiveness and implementation trials are a possible path to successful technology development and adoption.

## 1. Introduction

Chronic neurological, musculoskeletal, and cardiopulmonary conditions, as well as diabetes, are among the leading causes of disability among middle-aged and older adults globally [1]. Physical rehabilitation is an evidence-based approach to treating and managing the impairments in motor control, muscle paralysis, weakness, deconditioning, altered sensation, brain–body–nervous system dysregulation, and limitations in the activities of daily living. Intensive skilled physical rehabilitation, comprising progressive strengthening and functional mobility training, may be used for all these conditions to improve clinical outcomes. However, healthcare systems globally have issues with availability, insurance coverage, and accessibility for outpatient and home health services. This may cause chronic disease care to be either episodic with short durations or scarcely available at all. A significant portion of disease management relies on the individuals’ continued success with adherence to behavior changes and movement strategies learned in rehabilitation at home. The hope is that people acquire the skills and knowledge they need to incorporate evidence-based treatments into their daily lives. However, many people are not able to engage in evidence-based movement strategies at the levels needed for clinically meaningful outcomes in a home setting [2,3] and adherence to recommended strategies is known to be low. Consequentially, most people with chronic conditions decline in function as they live with the condition over time [2,4]. A model of care that promotes engagement in high-intensity therapeutic approaches at home could have significant impacts on adherence, independence, and quality of life.

Technology could help bridge the gap between intensive treatment in a clinic and at home. Rehabilitation technologies such as robotic exoskeletons, virtual and augmented reality systems, and remote monitoring systems have seen unprecedented growth and advancements in rehabilitation research over the past decade. Robotic exoskeletons are mechanical devices that can augment the functioning of the human body by supplementing existing movement, fully replacing non-existent movement [5], or improving balance and gait impairments [6,7,8]. Virtual reality (VR), a “technology that simulates a 3D virtual world and allows users to interact with a virtual world through computer simulation which senses the user’s state and operation and replaces or augments sensory feedback information to one or more senses in a way that the user gets a sense of being immersed in the simulation” [9], could be a tool for balance and fall prevention [10,11,12]. Augmented reality (AR), a technology that superimposes a computer-generated image onto an image in the real world, allowing interaction between virtual images or objects and real-world images, used therapeutically, could also augment rehabilitation [13]. Remote monitoring with wearable sensors collects real-time movement characteristics at home with the intent to provide meaningful information on health and function [14].

These technologies hold great promise to improve patient care by supporting the performance of evidence-based therapeutic activities and have the potential to be utilized in a patient’s home. However, the adoption of these technologies to augment therapeutic approaches has challenges. Historically, the adoption of evidence into clinical practice is fraught with obstacles with an average of 17 years between clinical trials and adoption into routine practice, and with only half of efficacy research adopted [15]. From a technological perspective, this poses monumental challenges as technology evolves at a pace that far outreaches the time needed to adopt evidence into practice. In other words, technology that is found to be efficacious may be obsolete by the time it is adopted into practice. This current gap between research and practice limits the impact of technology on improving outcomes, while costing billions of dollars in research and development to be rarely used in patient care.

In this narrative review, we focus on three rehabilitation technology domains that have been extensively researched over the past two decades: (i) robotic exoskeletons, (ii) virtual and augmented reality systems, and (iii) remote monitoring with wearable sensors. We review the three main components of technology uptake in rehabilitation practice: first, we will establish the clinical need within the populations of interest for rehabilitation technology applied in the home and community settings; second, we will review the technology and current evidence of its efficacy; and lastly, we will discuss obstacles, implementation needs, and suggestions for future research.

## 2. Materials and Methods

For this narrative review, we aimed to identify the highest level of evidence in evaluating the efficacy of clinical outcomes. Aligned with the goals for narrative reviews, we conducted a broad search in Pubmed and Cochrane systematic reviews. To capture the state of the literature on the efficacy of these rapidly growing technologies, we limited studies to publications between 2018 and 2023. Systematic and meta-analytical reviews were initially identified, with randomized controlled trials (RCTs) included when systematic reviews were limited. The articles identified in the literature search were reviewed to meet the inclusion and exclusion criteria and appropriateness for our study aims. The reference lists of the included articles were searched for additional studies. The included articles were qualitatively synthesized. Inclusion criteria were publications (i) available in the English language, (ii) pertaining to technologies that met the definitions of exoskeletons, VR, AR, or mHealth applications with remote monitoring intervention, and (iii) including an adult population (aged 18 or older). Additionally, the technologies needed to be evaluated in the context of physical rehabilitation or movement outcomes for neurological conditions (i.e., stroke, Parkinson’s disease (PD), multiple sclerosis (MS), vestibular disorders, spinal cord injury (SCI)), musculoskeletal conditions (i.e., chronic pain and chronic arthritis), heart disease, pulmonary disease, and diabetes.

Exclusion criteria were non-English articles, technology development or pilot tests, editorials and observational studies, and pediatric populations.

Search terms: exoskeleton (Exo* OR exoskeleton OR Robotic), virtual reality/augmented reality (Virtual Reality OR Exergam* OR Augment* Reality*), remote monitoring: (remote monitor* OR wearable sens*), rehabilitation (physical therapy OR Rehab*)

## 3. Clinical Need in the Populations of Interest

We examine the use of augmented technology in five populations of interest: (i) chronic neurological, (ii) chronic musculoskeletal, (iii) diabetes, (iv) chronic obstructive pulmonary disease, and (v) chronic cardiovascular disease. These five populations comprise the leading disabling conditions that utilize rehabilitation services [1]. Of the chronic neurological conditions, we focused on stroke, Parkinson’s disease, multiple sclerosis, and vestibular conditions; of the chronic musculoskeletal conditions, we focused on chronic pain and arthritis; and of the chronic cardiovascular diseases, we focused on heart disease. Patients with these conditions are commonly treated with physical therapy. Each of these populations has unique clinical needs that present the opportunity for augmentative technologies as described below.

### 3.1. Neurological Conditions: (e.g., Stroke, Degenerative Conditions, Spinal Cord Injury, and Vestibular Disorders)

Neurological conditions are the leading cause of disability-adjusted life years globally, and this is expected to increase as the population grows and ages [16]. People with neurological conditions experience impairments in neuromuscular control, resulting in paresis, altered muscle tone, muscle weakness and decreased endurance, and impaired balance, which in turn results in difficulty standing, walking, transfers, performing self-care activities, and engaging in personal and social role activities. Therapeutic rehabilitation can improve motor control, muscle performance, balance, gait, and activity and task performance; however, therapeutic dosing needs to be at high intensity with task-specific repetition and progressive loading and challenge [17,18,19,20,21]. In a clinical setting, this is easily monitored by a therapist, but implementation and adherence in a home setting is difficult. Implementation and adherence are limited due to issues such as access to affordable and accessible programming, lack of specialty equipment, difficulties finding transportation or caregiver assistance, cost [22], fear of falling [23], and fatigue [24]. As a result, many patients with neurological conditions experience a decline in activity levels in a home setting and poor compliance with therapy recommendations long-term [3].

### 3.2. Musculoskeletal Conditions (e.g., Chronic Pain and Arthritis)

Approximately 1.3 billion people worldwide suffer from musculoskeletal conditions, with the highest prevalence of low back pain, osteoarthritis, and neck pain [25]. Chronic pain and conditions such as arthritis, a condition also marked by pain, result in stiffness and limitations in movement and daily tasks. Physical inactivity is common. Over 66% of people with knee and hip osteoarthritis do not engage in the recommended levels of exercise and 80% do not engage in a regular strengthening exercise program [26]. Walking and strengthening exercises are well established as effective treatments [27], yet adherence to these treatments is poor [2,28,29].

### 3.3. Metabolic Conditions (e.g., Diabetes)

Diabetes occurs when the pancreas does not produce enough insulin or does not process insulin properly. Diabetes is estimated to affect approximately 415 million people worldwide, with a prediction to grow to over 600 million in the next 10 years [30]. Poorly managed diabetes can lead to devastating health impacts such as death, blindness, amputations, and chronic renal disease. Successful management of this diagnosis includes addressing a combination of modifiable risk factors with targets at improving nutrition, physical activity, and medication adherence. Rehabilitation services promoting physical activity are crucial for the management of diabetes as physical activity can improve hemoglobin A1c levels, insulin sensitivity, and glucose uptake [31]. It is well known that changes in the self-management of health behaviors can improve long-term health outcomes for this population, but creating sustained behavior change is challenging [32]. Targeted interventions that are sustainable and can improve long-term self-management for this patient population are crucial.

### 3.4. Pulmonary Conditions (e.g., Chronic Obstructive Pulmonary Disease)

Over 500 million people worldwide are estimated to have a chronic respiratory condition, with chronic obstructive pulmonary disease (COPD) as the leading cause of disability within these conditions [33]. COPD is currently not a curable condition, but there are many long-term self-management options that can improve outcomes and decrease the risk of exacerbations. Exacerbations of COPD are extremely costly on healthcare utilization and can lead to hospitalization, mortality, and decreased quality of life. Pulmonary rehabilitation has been shown to help reduce the risk of exacerbations and improve quality of life for patients with COPD [34]; however, pulmonary rehabilitation is largely underutilized. This is due to the lack of access to pulmonary rehabilitation clinics, with less than 3% of people with COPD accessing pulmonary rehabilitation globally. Access is significantly low in rural areas and developing countries due to a lack of outpatient rehabilitation clinics. Telerehabilitation is a promising option for improving access to pulmonary rehabilitation but requires significant implementation efforts to make a meaningful change in access [35].

### 3.5. Cardiovascular Conditions: (e.g., Heart Disease)

Cardiovascular disease is the leading cause of disease burden in the world and is estimated to be present in over 500 million people globally [36]. Cardiac rehabilitation is an evidence-based practice used to decrease the risk factors and mortality of cardiovascular disease, as well as to improve function and quality of life. The main components of cardiac rehabilitation include nutritional counseling, weight management, vitals management, physical activity counseling, exercise, and lifestyle modifications. The final phase of cardiac rehabilitation is aimed at creating independence and self-management of this chronic condition. While there is strong evidence to support formal cardiac rehabilitation programs, participation and adherence to these programs are low. The main limitations for participation in cardiac rehabilitation include lack of insurance coverage, poor motivation, limited program accessibility, lack of time, and fear of exercise [37].

## 4. Technology Review and Efficacy

### 4.1. Robotic Exoskeletons

#### 4.1.1. Technology Features

Robotic exoskeletons augment the movement system by providing supplemental forces and mechanics to body segments via an external, powered, motorized orthosis to move limbs [5,38]. These devices comprise hardware with rigid shanks and mechanical joints and are worn on the body. Software is embedded into the system with the goal of approximating normal human movement. Systems may be single-joint (ankle, knee, or hip) or multi-joint and can support both static and dynamic conditions [6].

Newer research embeds muscle sensors and stimulators into systems for more efficient movement control [39]. These human–machine coupling models with actuators are designed to replicate the neuromechanical elements of human movement [39]. The sensors can ascertain changes in task performance, muscle strength and fatigue, and environmental features, which may enable greater support for walking on diverse terrains [39,40]. Research is also expanding into the upper extremity, back, and ankle/foot exosystems [40,41], and more flexible systems with softer components than the traditional hard mechanical components of robotic devices [42]. Flexible systems are lighter and have less bulk, but it is not clear whether these systems can support the full weight of the lower extremities and body posture. These lighter systems may be useful for alternative options such as upper extremity functions [41,42].

#### 4.1.2. Efficacy

Among people with stroke, two meta-analyses (one of 13 studies with 492 participants and another of 20 trials with 758 participants) show small to moderate effects of exoskeleton training on walking and balance compared to conventional training [7,8] (See Table 1 and Table S1). The length of chronicity of stroke varied from 1.2 months to 6.5 years. The intervention times ranged from 1 to 40 sessions, with a duration of 10 days to 10 weeks. The total training time ranged from 150 min to 1800 min (about 1.5 days) and 20 min to 120 min per session. The frequency of sessions ranged from 2 to 5 times per week. All treatments were clinic-based, and there were no adverse events. A quality assessment of the articles ranged from fair to high. In two systematic reviews of stroke patients using exoskeletons for gait training, some evidence was found to support the use of exoskeletons; however, the findings in these two studies were synthesized with qualitative methodology [6,43].

In PD, two systematic reviews of the use exoskeletons for postural control and gait show improved balance and gait parameters; however, the meta-analytical outcomes were not reported [44,45]. The quality of study designs and interventions was generally low and heterogeneity was noted. Training with exoskeleton devices ranged from 10 to 20 sessions, 2–5 times a week, for 3–5 weeks. Among people with MS, use of an exoskeleton improved walking velocity and endurance, mobility, balance, and fatigue [46], with some evidence showing exoskeleton treatment is similar to conventional approaches in people with mild to moderate severity of MS and possibly superior to conventional methods among people with severe disease [47].

Among people with SCI, Dijkers et al. [48] published a systematic review of systematic reviews examining applications of exoskeletons to gait-assisted walking among people with spinal cord injuries and other neurological conditions. Of the systematic reviews, 82 percent pertained to people with spinal cord injuries, and the rest included mixed neurological populations. An overall poor study quality was noted. The authors concluded there was insufficient evidence to support the use of exoskeletons at home and in the community and to provide impact on daily life activity outcomes. In a 2018 systematic review of 12 studies (521 subjects), no improvements in gait speed compared to conventional training were found and low study quality was noted [49]. Conversely, a 2023 systematic review carried out by Zhang et al. reported improvement in gait but was also limited by low study quality and heterogeneity [50].

Evidence for the use of exoskeletons for chronic musculoskeletal applications is limited. In one study of 24 adults in a home setting, an ankle-, knee-, and hip-powered exoskeleton did not immediately improve physical performance; however, over time, with additional use at home, stair time and knee pain significantly improved [51]. Similarly, evidence for exoskeleton use for patients with diabetes, pulmonary conditions, and heart disease is limited. There were no randomized controlled trials or systematic reviews on the effectiveness of exoskeleton use for any of these chronic conditions.

**Table 1:** Efficacy of exoskeletons in rehabilitation outcomes in adults with disabling conditions.

Author	Study Design	Population	Intervention	Outcome
Stroke				
Calafiore et al. [43]	Systematic review	Stroke (*n* = 14 studies, 576)	Lokomat end-effector trainer and exoskeleton	Two studies of exoskeletons showed improvements in walking; poor to good quality studies
Hsu et al. [7]	Systematic review with meta-analysis	Stroke (*n* = 13 studies; 492 subjects) 1.2–75 months post-stroke Mixed ambulatory status	Exoskeleton-assisted training compared to dose-matched conventional training 10–40 sessions, 10 days–10 weeks; 150–1800 min	Improved outcomes with exo-training: walking speed [MD] 0.13 m/s, 95% CI (0.05; 0.21), balance [SMD] 0.3, 95% CI (0.07, 0.54); after follow-up, mobility [SMD] 0.45, 95% CI (0.07, 0.84) and endurance [SMD] 46.23 m, 95% CI (9.90, 82.56)
Karunakaran et al. [6]	Systematic review	Adults with acquired brain injury (*n* = 45 studies of stroke); total subjects not reported)	Lower extremity robotic exoskeleton for overground walking 1–40 sessions	Generally improved balance gait characteristics; motor impairments but some inconsistencies; no effect on global disability or spasticity
Leow et al. [8]	Systematic review with meta-analysis	Stroke (*n* = 20 studies; 759 subjects)	Exoskeleton-assisted training compared to conventional training 2–5x/week; 10 days–10 weeks 20–120 min/session	Improved exo-training: walking ability (d = 0.21; 95% CI (0.01–0.42)); follow-up (d = 0.37; 95% CI (0.03, 0.71)); and walking speed (d = 0.23; 95% CI (0.01–0.46)) Nine studies examined follow-up 22 weeks–12 months
Parkinson’s Disease				
Carmignano et al. [44]	Systematic review	Parkinson’s disease (*n* = 20 studies)	Exoskeleton-assisted training (*n* = 9 studies); end-effector robots (*n* = 11 studies) 2–5 sessions per week; 3–5 weeks; 20–45 min	Improved gait but not superior to conventional treatment, except in more severe disease No adverse outcomes Quality appraisal low to high
Picelli et al. [45]	Systematic review	Parkinson’s disease (*n* = 18 studies);	Exoskeleton-assisted training *n* = 9 studies; end-effector robots (*n* = 9 studies) 10–20 sessions over 2–5x/week, 20–40 min duration per session	Improved balance and less freezing of gait Moderate quality studies
Multiple Sclerosis				
Bowman et al. [46]	Systematic review	Multiple sclerosis (*n* = 12 studies)	Exoskeleton (*n* = 10 studies) 6–40 sessions, 2–5 sessions, 3–8 weeks	Improved balance and gait Fair to good study quality, 65% good to excellent study quality
Calabro et al. [47]	Systematic review	Multiple sclerosis (17 studies)	Lokomat grounded robotic device (*n* = 13), power exoskeleton (*n* = 2), end-effector (*n* = 2) 2–5 sessions/week, 3–18 weeks	Improved gait speed, balance, and endurance; more severe patients improved functional outcomes when paired with VR; improved spasticity, fatigue, pain, psychological wellbeing, and quality of life
Spinal Cord Injuries				
Hayes et al. [49]	Systematic review	Spinal cord injuries (*n* = 12 studies; 521 subjects) ASIA scores A-D	Exoskeletons (*n* = 3), Locomat (*n* = 9) 11–41 sessions, over 4–24 weeks	No improvements in gait speed compared to conventional training; low to high study quality
Zhang et al. [50]	Systematic review	Spinal cord injury (*n* = 11 case-control studies) Complete and incomplete injury ASIA 1A–5A	Power exoskeletons 2–5 sessions/week 30–90 min 1 month–1 year	Improved gait; low study quality and hetereogeneity
Mixed Neurological Conditions				
Dijkers et al. [48]	Review of systematic reviews	Neurologic populations (*n* = 17 studies) (stroke, SCI)	Powered exoskeletons for gait training Intervention dosing not provided	Systematic reviews poor-quality; failure to report important clinical characteristics; caution is warranted for decisions on technology use
Musculoskeletal				
McGibbon et al. [51]	Randomized control trial	Knee osteoarthritis (*n* = 24 subjects two-stage cross-over design)	Exoskeleton in clinic and home training 2-week use of device at home; 2 weeks of non-use	No immediate effects; improved stair time (*p* = 0.001), WOMAC pain (*p* = 0.004), and function (*p* = 003)
Diabetes/COPD/Heart Disease				
No studies found				

MD: mean difference; SMD: standardized mean difference; d: Cohen’s d effect size; WOMAC: Western Ontario and McMaster University Arthritis Index; ASIA: American Spinal Injury Association Scale.

### 4.2. Virtual and Augmented Reality

#### 4.2.1. Technology Features

VR and AR technologies are advancing rapidly and are increasingly directed at healthcare use. VR is a technology that simulates a 3D virtual world and allows users to interact with the virtual world in a manner similar to reality [9]. AR technology superimposes a computer-generated image onto an image in the real world and allows interactions between virtual images or objects and real-world images [52].

There are four basic elements of VR: the virtual environment, virtual presence, sensory feedback, and interactivity. It is a powerful modality that may drive neuroplasticity by providing a high number of repetitions, permitting quick and small alterations in task difficulty, and keeping participants motivated and engaged during training sessions [53]. There has been growing interest over the last decade in the potential of VR to promote motivation for exercise [10], behavior change [54], rehabilitation of motor skills [55], and fall prevention [55,56]. VR includes approaches designed for rehabilitation applications and those that are generic, game-based, or exergaming approaches (e.g., Nintendo Wii, Xbox, and PlayStation) [57].

#### 4.2.2. Efficacy

There were 16 systematic reviews examining the effects of VR, AR, or mixed reality, with the majority examining VR or mixed reality approaches in rehabilitation [10,11,12,58,59,60,61,62,63,64,65,66,67,68,69]. (See Table 2). There was one study that examined AR-only interventions [70]. There were seven studies comprising neurological patients (vestibular dysfunction (*n* = 3) [58,59,60], stroke (*n* = 1) [11], stroke and older adults with balance deficits (*n* = 1) [70], stroke and PD (*n* = 1) [10], and PD (*n* = 1) [12] (See Table 2)). The intervention dosing ranged from 4 to 120 min sessions at a frequency of 1–7x/week with a duration anywhere between 1 week and 6 months.

In people with vestibular dysfunction, VR interventions compared to conventional treatments improved dizziness inventories [58,59,60]. There was one study that showed the beneficial impact of VR on balance [60], whereas no effects were noted on Activities-Specific Balance Confidence [58,59,60]. For people with stroke, the largest study (*n* = 12 studies, 350 subjects) showed a small beneficial effect on function, gait, and balance [11]. A study of stroke and older adults comprising four RCTs showed a small effect on function but no effect on balance [70]. Another study of people with stroke or PD showed no difference between VR intervention and conventional treatment for outcomes, but that motivation may be improved with VR interventions [10]. For people with PD, balance improved with VR; however, there was no improvement in gait, activities of daily living (ADLS), motor function, or quality of life [12]. These studies had high heterogeneity with low to high risk of bias.

There were five studies comprising people with chronic musculoskeletal conditions: four were of pain populations [62,63,64,65] and one consisted of people after total knee replacement [61]. All the studies examined the effects of VR. Pain improved in all studies of pain populations [62,63,64,65]. Balance improved among people with total knee joint replacement, however, VR did not improve function [61]. On the other hand, function and disability improved in two studies [64,65].

Equally, one randomized control trial examined the effects of VR on diabetes management [71]. The results from this study showed that VR had significant effects on blood glucose levels and muscle mass when compared to a control group. However, there was no significant impact on body mass index.

For pulmonary conditions, one study examined VR outcomes among patients. Among people with COPD participating in a randomized control trial, the use of VR with cardiopulmonary rehabilitation improved strength, functional mobility, and walking endurance [72].

There were three systematic reviews that examined the use of VR and exergaming among people with heart disease [66,67,68], and two systematic reviews using qualitative methodologies reported the use of VR improving pain and heart rate, energy levels, walking endurance, adherence, and motivation [67,68]. However, a systematic review using meta-analytical approaches reported no statistical differences between the VR and control groups, and high heterogeneity was noted [66].

### 4.3. Remote Monitoring

#### 4.3.1. Technology Features

Remote monitoring, a form of telehealth that allows monitoring of patient performance outside of the clinic to capture movement and health trends in a real-life context, is typically conducted using smartphone applications and/or embedded sensors (e.g., in phones or wearable devices). The most common wearable sensors are motion sensors (e.g., accelerometers and gyroscopes) or biopotential sensors (e.g., ECG or EMG) [73]. Sensors can provide a wide variety of information, including activity levels, vital signs, posture, range of motion, respiration, falls, pressure/loading information, temperature, and gait characteristics [74]. Remote monitoring can be used passively to track trends, or can be utilized as an intervention by pairing it with feedback, reminders, gaming, financial incentives, and provider messaging as ways to promote engagement.

#### 4.3.2. Efficacy

Overall, 13 studies examined the results of remote monitoring on rehabilitation. The type of intervention and dosing were widely variable. Approaches to intervention included both remote monitoring to augment telehabilitation services and promoting improved self-monitoring for behavior changes. The frequency of monitoring ranged from continuous to only during treatment sessions, with feedback from a healthcare professional ranging from real-time to 1x/month. The duration of monitoring ranged from 11 days to 18 months. There are few studies on the impact of remote monitoring interventions for the chronic neurological population on rehabilitation outcomes. There were two systematic reviews on the use of remote monitoring intervention in the neurological population [75,76] and one RCT [77] (Table 3). Among people with stroke, one systematic review of four studies showed wearable sensors had no effect on step count [75]. Similarly, in a randomized controlled trial, remote monitoring with feedback did not increase walking time but was found to be useful for therapists [77]. There was one systematic review of people with PD, with a meta-analysis of two studies, showing no improvement on balance and function compared to standard care, but did show improvements on quality of life and adherence [76]. No efficacy studies (systematic reviews or randomized controlled trials) were identified for people with MS. Remote monitoring shows no greater improvement to standard care in the neurologic population in terms of activity (i.e., walking activity level, balance, etc.) [75,76,78], but shows initial promise for improvement in quality of life and adherence with this treatment approach [76].

The efficacy of remote monitoring for chronic musculoskeletal conditions is limited. (See Table 3). For Christiansen et al. [78] in an RCT of 43 subjects recovering from total knee joint replacements, the use of a Fitbit sensor with a plan for goal setting and a monthly phone call resulted in a small significant increase in activity in the intervention group. On the other hand, another study showed no effects on activity after knee and hip surgery but showed remote monitoring decreased hospital readmission [79].

The use of remote monitoring in diabetes care is examined in one systematic review with a meta-analysis (Table 3). The systematic review and meta-analysis showed that the use of telemonitoring devices was beneficial at reducing HbA1c and weight compared with usual care. Subgroup analysis suggested telemonitoring with and without real-time feedback improved outcomes compared to other monitoring approaches [80].

In COPD, remote monitoring has been mostly used in the context of facilitating pulmonary rehabilitation programs in the home. Evidence on its use is mixed. There were three RCTs identified. (Table 3). Remote monitoring in conjunction with an exercise program resulted in an improvement in quality of life [81] and lower incidence of hospitalization, and improvements in strength and health status [82]. Conversely, another RCT found remote monitoring had no improvement on symptoms, healthcare use, and self-management [83].

For heart disease, three systematic reviews and one RCT were identified (Table 3). Evidence shows that remote monitoring and telerehabilitation can improve exercise capacity, systolic BP [84], functional capacity, step count, exercise habits, and depression scores compared to usual care [85]. Additionally, evidence shows that remote monitoring can improve self-care behaviors [86]. There is mixed evidence about the ability for remote monitoring to improve quality of life [84,85]. Studies showed that home-based cardiac rehabilitation and clinic-based rehabilitation were comparable [85,87]. The results of one RCT included a cost comparison between remote cardiac rehabilitation programs and in-clinic care and found that per capita, program delivery and medication costs were lower for the remote monitoring group, but there were no statistically significant differences in hospital service utilization costs [87].

Overall, current evidence of the effectiveness of remote monitoring is limited in the neurologic, musculoskeletal, and diabetes populations; however, there is evidence that remote monitoring combined with therapeutic programming is comparable to traditional rehabilitation in cardiac rehabilitation and has significant benefits over usual care. However, the long-term outcomes need to be seen to better understand the impacts on long-term self-management, adherence, and implementation factors such as cost comparisons and uptake.

**Table 3:** Efficacy of remote monitoring with sensors on rehabilitation outcomes in adults with disabling conditions.

Author	Study Design	Population	Intervention	Outcome
Neurological Conditions (Stroke and Parkinson’s Disease)				
Stroke				
Dorsch et al. [77]	Randomized control trial	Stroke (*n* = 135); 11 countries, in-patient acute care setting	Wearable sensors for walking; effects of quantitative feedback on daily walking Monitoring throughout day, 5–7 days/week, with 3x/week feedback, duration mean of 22.5 days	No effect: augmented feedback did not increase time walking, but was useful for therapists
Lynch et al. [75]	Systematic review	Stroke (*n* = 4 studies, 245 subjects), time post-stroke 1 week to 51 months	Wearable sensors Variable monitoring rate, inconsistent reporting on time and frequency 30 min–1 h/day, 3x/week, duration 11 days–12 weeks	No effect on step count; low-quality evidence
Parkinson’s Disease				
Ozden et al. [76]	Systematic review and meta-analysis	Parkinson’s disease (*n* = five studies, two in meta-analysis)	Varied approaches (mobile apps and/or sensors) Inconsistent reporting frequency and time, duration 6 weeks–12 months	Sensors plus apps were equivalent to standard treatment in balance and function Sensors plus apps improved quality of life and adherence compared to standard treatment. Good quality studies with low–high risk of bias
Musculoskeletal Conditions				
Christiansen et al. [78]	Randomized control trial	Total knee replacement (*n* = 43 subjects)	Fitbit, step goals, and one phone call with standard care Continuous monitoring with one call/month, duration 6 months	Intervention increased step count, time walking, and engagement in moderate to vigorous physical activity compared to control
Mehta et al. [79]	Randomized control trial	Hip and knee arthroplasty (*n* = 147 subjects)	Wearable activity monitoring Daily monitoring and messaging 1–3x/week. Duration 45 days	No effects on discharge, return to activity; remote monitoring decreased readmission
Diabetes				
Michaud et al. [80]	Systematic review + meta-analysis	Diabetes (*n* = 17 studies, 15 studies for meta-analysis)	Telemonitoring devices Duration 3–12 months	Small reduction in HbA1c and weight loss compared to usual care. Subgroup analysis suggested telemonitoring with automatic mobile transmission or with real-time feedback modality may lead to a greater improvement in HbA1c outcomes when compared with telemonitoring without these features. Low- to high-quality
Chronic Obstructive Pulmonary Disease (COPD)				
Benzo et al. [81]	RCT	COPD (*n* = 375)	Home-based rehabilitation with remote monitoring and health coaching intervention Continuous monitoring with weekly calls. Exercise included three exercises daily, 6 days/week. Duration 12 weeks	Significant and clinically meaningful difference between the intervention and control in terms of physical and emotional disease specific quality of life scores (95% confidence interval): 0.54 points (0.36–0.73), *p* < 0.001; 0.51 (0.39–0.69), *p* < 0.001.
Zanaboni et al. [82]	RCT	COPD (*n* = 120)	Telerehabilitation and treadmill training with self-management website that is remotely monitored by physiotherapists. Three groups: (1) Telerehab and treadmill program supervised by PT (2) unsupervised treadmill training with exercise diary, booklet, and individualized training program, and (3) usual care Weekly monitoring, 30-min sessions 3–5 times/week exercise Moderate- to high-intensity. Duration 8 weeks	Significant decrease in hospitalizations and emergency department visits in intervention groups compared to usual care. Significant improvement in COPD health status (*p* = 0.002) and strength compared to control group (*p* = 0.027). No differences between groups were detected for self-efficacy, anxiety, and depression scores.
Stamenova et al. [83]	RCT	COPD (*n* = 122)	Three groups: (1) remote monitoring by respiratory therapist, (2) self-monitoring, and (3) standard care Monitoring: oxygen saturation, blood pressure, temperature, weight, and symptoms Duration 6 months	No significant difference between groups in self-management, knowledge, symptoms, or healthcare use.
Heart disease				
Zhong et al. [84]	Systematic review with meta-analysis	Percutaneous coronary surgery recovery (*n* = 5 studies, 585 subjects)	Home-based cardiac telerehabilitation with remote monitoring 1–5x/week, inconsistent reporting of time and intensity, duration 6–24 weeks	Improve physical exercise capacity (6WMT), systolic blood pressure, triglycerides, and low-density lipoprotein cholesterol compared to control. No effect on quality of life, diastolic blood pressure, total cholesterol, and high-density lipoprotein cholesterol. High variability of intervention models
Ramachandran et al. [85]	Systematic review + meta-analysis	Heart disease (cardiac rehab phase 2) (*n* = 14 studies, 2869 subjects)	Home-based cardiac telerehabilitation. Three groups: (1) home-based cardiac telerehabilitation, (2) usual care, (3) clinic cardiac rehabilitation Session monitoring, 1–5x/week, duration 6 weeks–6 months	When compared with usual care, home-based cardiac rehab showed significant improvement in functional capacity, daily step count, exercise habits, depression scores, and quality of life (short-form mental component summary and physical component summary scores). Home-based rehab and clinic-based cardiac rehab were comparably effective on functional capacity physical activity behavior, hospitalizations, and quality of life. Variable risk of bias
Nick et al. [86]	Systematic review	Heart failure (*n* = 12 studies, 1923 subjects)	Telemonitoring for improving self-care behaviors Frequency of user interface with device varied between 2x/day and 1x/week for 2–18 months	Improved self-care behavior with use of telemonitoring. There is insufficient and conflicting evidence to determine how long the effectiveness lasts. Medium- to high-quality studies, low to mod risk of bias
Maddison et al. [87]	RCT non-inferiority trial	Heart disease (*n* = 162)	Remote telerehabilitation with monitoring Intervention: telerehabilitation exercise prescription, exercise monitoring (vitals, ECG, and accelerometry), coaching, and theory-based behavioral strategies. Control: Clinic-based in-session monitoring. Three sessions/week for 12 weeks. Ranged from 30 to 60 min sessions and 40%–65% heart rate reserve	Effect: Remote telerehabilitation program with monitoring was non-inferior to clinic-based rehabilitation program. Remotely monitored participants were significantly less sedentary compared to the control. Clinic-based participants had better improvements in waist and hip sizes compared to intervention. No other between-group differences were detected in VO2max, exercise adherence, motivation, or quality of life. Costs: The per capita program delivery and medication costs were significantly lower for the remote monitoring group. The hospital service utilization costs were not statistically significantly different between groups.

HbA1c: hemoglobin A1C; VO2max: maximal oxygen consumption; 6MWT: 6-min walk test.

## 5. Obstacles, Implementation, and Suggestions

### 5.1. Obstacles

#### 5.1.1. Patient/Clinician Needs: Lack of Research on Usability and Patient Centered Outcomes

Research is lacking on user experiences. Less than 10% of remote monitoring research explicitly examines patient experience and less than 2% measures clinician experience [88]. Studies that do assess patient experience primarily employ usability surveys, while focus groups or interviews that elicit a more in-depth understanding of patients’ perspectives are infrequently performed. A lack of user-friendliness, performance issues, and technical errors is reported as a barrier to the widespread use of technology [73]. Additionally, devices that are bulky and heavy are less likely to be adopted as they are difficult and time-consuming to don. Similarly, devices that are overly complicated and require significant specialty training to use pose barriers to home use. Other possible obstacles include physical space, lack of clinician time and expertise to train individuals, and a lack of personalization and ability to adapt to changes in health status [89].

#### 5.1.2. Effectiveness: Difficulty with Comparisons, Insufficient Research across Populations, Lack of Long-Term Outcomes

Across the board, the evidence of effectiveness in these technologies is lacking. A big challenge in technology is the lack of consistent reporting on the specific technological components, intervention dosing, and intervention components. Additionally, there is heterogeneity between studies in the outcome measures chosen and the specific subgroups of the patient population being tested (i.e., severity level, acuity, etc.). This combined with the wide variety of competitive types of different technology makes comparisons of technology studies difficult.

The majority of research on these technologies heavily focuses on the development phase, with emphasis on reliability, validity, and intial testing. Follow-up research is mostly focused on the use of the technology in clinic settings with scarce research performed to determine the efficacy of these technologies specifically in the home and community. Remote monitoring for pulmonary and cardiac rehabilitation is an exception to this and rehabilitation services in the home have more readily been researched here. Furthermore, little is known about the technologies’ long-term efficacy for sustained behavior change at home and in the community, including the promotion of activity and participation in social roles.

#### 5.1.3. Lack of Healthcare Service Outcomes

Service outcomes include areas such as the efficiency, timeliness, reimbursement, safety, and equity of the intervention. Of these service outcomes, safety in terms of adverse events was the only outcome assessed among studies in our efficacy review. Consideration of safety in a home setting is paramount for technology use in the home. Use of technology in both home and community settings means there is less supervision, and an individual is using technology in uncontrolled environments compared to a controlled clinic setting. Research is needed to ascertain the safety of these devices at therapeutic intensities in a home setting. Ethical and legislative concerns are also salient as the ability to track everyday movements at home calls for enhanced measures to ensure data privacy, security, and accountability for any adverse outcomes.

Other service outcomes are necessary to evaluate how technology can fit into current healthcare systems. Early evidence on most of these technologies demonstrates that there is a comparable effect on the standard of care. If the clinical outcomes are comparable, then researchers must start to consider what interventions are preferable based on clinician/patient needs (see above) and what will work best, most efficiently, and most equitably for sustained healthcare delivery.

#### 5.1.4. Lack of Implementation Outcomes and Strategies

Implementation outcomes are rarely assessed in technological research. This would include outcomes such as cost, the acceptability of an intervention to practictioners and clinicians, how feasibile adoption of the intervention is, and more. None of the reviewed studies commented on specific implementation strategies for adopting the technology into practice after the study period had ended. Cost is a major barrier to implementation for all technologies. However, while cost is commented on in most of the reviewed articles, only two of the included studies had cost comparison outcomes [87,90]. The estimated cost for an individual to have home use of an exoskeleton is anywhere from $70,000 to $100,000 [38], commercial VR solutions often exceed $5000 [9], and costs for remote monitoring are somewhere between $275 and $7963 USD per patient per year depending on what is being monitored. However, these costs can be anticipated to continue to decline over time as the technology becomes less costly [91]. Cost-effectiveness comparisons of remote monitoring technologies have been made in cardiac rehabilitation research. A study in the Netherlands said cost-effectiveness was comparable between a clinic-based and telerehabitation model [90]. Conversely, a different study in New Zealand found that the program delivery costs were significantly lower for remote programming versus clinic programming for cardiac rehabilitation [87]. Healthcare utilization costs will likely vary significantly based on country, insurance reimbursement, healthcare delivery costs, and such factors. Both of these studies only looked at healthcare utilization costs rather than patient-centered costs, including transportation, lost wages, etc., which will also be important to understand in terms of equity.

### 5.2. Implementation and Suggestions: A Path to Facilitated Adoptions through Hybrid Research

The path to the implementation of rehabilitation technologies into practice is complex. While changes in clinical practice behavior are slow and effortful, changes in technological advancement are rapid. Technologies may be obsolete if it takes decades to adopt them into practice. Implementation science, defined as the “scientific study of methods to promote the systematic uptake of research findings and other evidence-based practices into routine practice” [92], could be used to help us navigate this gap.

Once technology is developed and ready for efficacy testing, its efficacy can be evaluated while examining the alternative outcomes of its implementation and its readiness for adoption. Effectiveness–implementation hybrid study designs allow for aims that examine the efficacy of the technological innovation while examining the implementation outcomes in a real-world application context [93]. Rather than confirming the efficacy of an intervention in a highly controlled manner, hybrid studies aim to inform clinical or policy decisions by providing evidence for the adoption of an intervention into clinical practice. This study methodology has many practical applications and provides more rapid translation of research into clinical environments. It also provides the chance for iterative technology adaptation based on the needs of patients, clients, and healthcare systems. This is especially helpful in technology research where advances emerge at a significantly higher rate than implementation.

**Figure 1:**
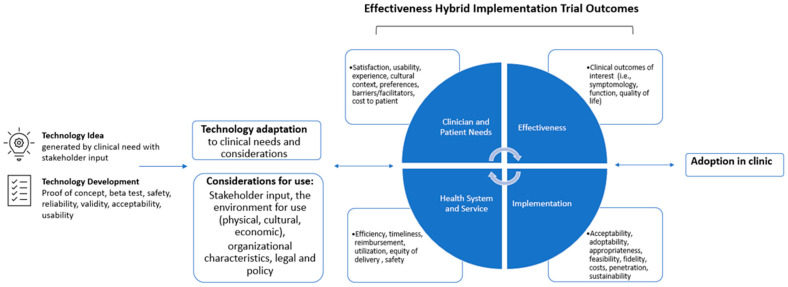
Conceptual model: a path to facilitated adoption through hybrid research and implementation science.

The suggested path of technological development to adoption is depicted in Figure 1. Figure 1 was created with combined components from Proctor et al.’s standards for implementation outcomes [94], the Consolidated Framework for Implementation Research (CFIR) [95], the Quality Improvement Framework (QIF) [96], and the Nonadoption, Abandonment, and Challenges to the Scale-up, Spread, and Sustainability of Health and Care Technologies (NASSS) framework [97]. The first phases of technological development start with stakeholder engagement. Partnerships between stakeholders—engineers, clinicians, and consumers (i.e., patients and families)—are a critical component that can facilitate adoption. Technology development should be closely aligned with stakeholder needs and values, and should be utilized to provide valuable insight on health systems and specific condition needs. Much of this information is not widely known to technology developers, and conversely, the opportunities and the availability of technology are often not known by clinicians and patients without the opportunity to engage with each other. Engineers, clinicians, and patients should collaboratively engage in all aspects of technology development and prototype testing. The next phase includes the preparation of technology for clinical use, including beta, safety, reliability, and validity testing. In preparation for clinical use, the team should revisit considerations including stakeholder input, the environment that the technology will be used in, cultural considerations, economic considerations, organizational characteristics, and any other legal considerations such as the HIPAA and privacy laws. We must address four areas of outcomes to perform a hybrid study on technology in clinical care: (i) clinician and patient needs, (ii) effectiveness, (iii) health systems and services, and (iv) implementation. Clinician and patient needs could include user-friendliness, the amount of assistance needed with the system, performance issues, users’ preferences, socio-emotional stigma concerns, technology acceptance, and the presence of technical errors (i.e., connectivity issues, motion artifacts, delayed processing speeds) [73]. Effectiveness would include typical clinical outcomes such as pain, function, quality of life, strength, etc. Effectiveness outcomes should also consider subgroup analysis to determine for which facets of the patient population the technology works best. The health systems and services outcomes include testing the efficiency of technology intervention compared to standard of care, healthcare utilization, the cost of administering the intervention, and service equity. Ethics and equity should be addressed and optimized throughout the process of development, testing, and adopting. Technology development and testing should incorporate different skin colors, environmental contexts, and mobility needs. The implementation outcomes include acceptability, adoptability, appropriateness, feasibility, fidelity of intervention protocol, costs, penetration, and sustainability for long-term use. These outcomes specifically focus on the factors that will impact regular use of the technology in the healthcare system and perform best within the defined implementation strategies and frameworks.

Lastly, implementation science supports an iterative approach to adopting any new evidence into practice. This includes revisiting after outcomes are evaluated to find new ways to improve and advance the technology to better suit the setting and clinician/patient needs.

## 6. Discussion

### 6.1. Summary of Findings and Comparison to Other Studies

The purpose of our narrative review was to discuss the clinical need, current evidence, obstacles, and implementation strategies for future research on three promising technologies that could be used to augment physical functioning in the home setting. All the main disease groups reviewed have needs for long-term self-management and are at risk of functional decline. The technologies reviewed hold promise to sustain long-term adherence to evidence-based rehabilitation treatments in a home setting; however, effective evidence is still limited.

Our review showed substantial literature pertaining to exoskeletons and VR among chronic neurological and musculoskeletal conditions, with limited applications of these technologies to other patient populations. Broadly, the use of exoskeletons in the neurological population has promise for improving gait, balance, and endurance, and VR improves dizziness among adults with vestibular disorders and may improve balance and mobility among people with strokes; however, the findings are mixed. Interestingly, Dijkers et al. [48] conducted a review of the systematic reviews of exoskeleton use among people with neurological conditions, with a focus on spinal cord injury. We concur with their findings that there is an important need for better study quality, outcome assessment, and the documentation of the clinical characteristic of study participants. Cardiac rehabilitation shows the most beneficial effect of the use of AR/VR and remote monitoring systems, and there is limited research on remote monitoring with sensor applications for other conditions.

All technologies have formidable obstacles to adoption. The main challenges highlighted from our review were a lack of focus on clinician and patient needs, including usability, satisfaction, and preferences; limited effectiveness research across populations due to significant heterogeneity in outcomes, reporting, dosing, and technological components, and lack of reporting; a lack of health system outcomes, including equity, efficiency, timeliness, and reimbursement; and a lack of implementation outcomes, including acceptability, appropriateness, cost, fidelity, penetration, and sustainability. We recommend the use of hybrid effectiveness and implementation studies to assess the outcomes in all these domains to increase the uptake of technology in practice. Consistent with our findings, a systematic review by Mattison et al. [88] on the effect of wearable devices on healthcare outcomes for chronic conditions found that there are limited studies investigating implementation, patient-centered outcomes, service outcomes, and economic outcomes. Mattison et al. also found that the outcomes in current research are focused on health outcomes rather than patient experience, clinician experience, and cost. Another systematic review carried out by Peyroteo et al. [98] also reiterates the major difficulty of remote technology is issues with integration into already existing systems and healthcare infrastructures. However, while this messaging is consistent in highlighting the problem of technology integration in practice as seen in many reviews, little is changing in emerging research and there is little guidance on how to integrate these outcomes in a practical way.

### 6.2. Contributions

To our knowledge, this is the first review to combine a broad overview of the major technologies being utilized across the most prevalent chronic diseases worldwide. It is also one of the first to make meaningful suggestions and guidance on the implementation of rapidly changing technologies with hybrid effectiveness and implementation models. The World Health Organization (WHO) has outlined the primary goals for Rehabilitation 2030 [99], which include the objectives of (1) strengthening rehabilitation planning and implementation and (2) building comprehensive rehabilitation service delivery models to progressively achieve equitable access to quality services, including those in rural and remote areas. This narrative review directly addresses these objectives by outlining implementation strategies to improve the integration of new research into practice. The focus of this paper also addresses the crucial issues of access and equity by investigating how technology can augment rehabilitation services in all areas by facilitating rehabilitation services at home.

### 6.3. Limitations and Conclusions

Our narrative review approach did not comprise the same level of rigor that is expected for systematic reviews, and as such, there are likely missed articles. Our approach was to undertake a broad descriptive review of technologies that have the potential to augment rehabilitation in a home setting in order to understand the nature of the field. A more in-depth critical review is warranted in several areas of this narrative review. Another limitation is that the scope of this review did not include all chronic disease populations. This was because we aimed to target the disease populations impacted by movement dysfunction the most and present most prevalently in rehabilitation clinics. Future research should evaluate other populations as well.

Overall, the future of using rehabilitation technology to foster and track movement at home is promising; however, a lot of work remains. There are many applications across patient populations, and improving technology implementation has significant potential to bridge the gap between the home and the clinic and to foster long-term change. However, the current path of technology development and testing may be insufficient to integrate technology into practice. The focus must be shifted in technology research to include community partnerships early in development and utilize research methodology that integrates implementation, service, and equity factors. The notion of using rehabilitation technologies to bring rehabilitation approaches into the home is exciting, but will we sink or swim in this rapidly evolving field?

## Figures and Tables

**Table 2:** Efficacy of virtual reality (VR) and augmented reality (AR) on rehabilitation outcomes in adults with disabling conditions.

Author	Study Design	Population	Intervention	Outcome
neurological (stroke, parkinson’s disease, vestibular disorders)				
Chu et al. [58]	Systematic review and meta-analysis	Vestibular disorders (*n* = 20 studies, 968 subjects)	VR Mixed home and supervised sessions 4–45 min sessions, 1–6 days/week, 3–12 weeks	Improved Dizziness Handicap Inventory [SMD]: −7.09, 95% CI: [−2.17, −2.00]); no effect on Activities-Specific Balance Confidence scale High heterogeneity
Heffernan et al. [59]	Systematic review and meta-analysis	Vestibular Dysfunction (*n* = 5 studies, 204 subjects)	VR and AR 20–45 min, 4–6 weeks	Dizziness Handicap Inventory SMD 1.13 (95% CI, −1.74, −0.52); high risk of bias noted
Mohamed Hazza et al. [60]	Systematic review and meta-analysis	Vestibular dysfunction (*n* = 6 studies, 258 subjects)	VR 10–45 min, 2–7 days/week, 1–6 weeks	Balance MD −3.27 (95% CI −4.27, −1.84); Dizziness VAS MD 25.13 95% CI (12.96, 37.29); Dizziness Handicap Inventory MD −12.93, 95% CI (−24.18, −1.69); No effect Activities-Specific Balance Confidence or Vertigo Analogue Scale
Demeco et al. [11]	Systematic review	Stroke (*n* = 12 studies; 350 subjects)	VR 30–120 min, 2–5x/week, 2–9 weeks	Improved upper limb function, gait, and balance. High heterogeneity.
Gil et al. [70]	Systematic review and meta-analysis	Stroke and older adults (*n* = 11 studies, 4 studies for meta-analysis, 308 subjects)	AR 20–120 min, 1–5x/week, 1–12 weeks	No effect balance (−1.12 (95% CI −3.54, 1.31), small effect on function (Timed up and Go) 92.81 (95% CI 1.04, 4.58)
Sevcenko et al. [10]	Systematic review	Stroke (*n* = 10 studies) Parkinson’s disease (*n* = 8 studies), 1052 subjects	VR 2–7x/week, 2–6 weeks	May be as effective as conventional treatments and may be more motivating. Good to high study quality
Kwon et al. [12]	Systematic review and meta-analysis	Parkinson’s disease (*n* = 14 studies, 524 subjects)	VR 30–60 min, 2–5 days/week, 6–12 weeks	Improved balance (Berg Balance Scale (MD = 2.71 95% CI 1.45–3.96) and Balance Confidence scale (MD = 9.43, 95% CI 5.67–13.19)); no improvement in gait, ADLs, motor function, or quality of life; risk of bias low to high; heterogeneity low to moderate
Musculoskeletal				
Blasco et al. [61]	Systematic review	Total knee replacement rehabilitation (*n* = 6 studies; 312 subjects)	VR with rehabilitation 20–60 min, two to seven sessions/week, 6–48 weeks	Improved balance; no improvement on function, pain, or satisfaction
Choi et al. [62]	Systematic review and meta-analysis	LBP (*n* = 11 studies, 1761 subjects)	Virtual-reality-based rehabilitation Intervention details not identified	Pain: small to medium effect (SMD = +/− 0.37, 95% CI (0.75 to 0.00) Low bias, high heterogeneity
Youssef et al. [63]	Systematic review	Orthopedic patient subjects (*n* = 19 studies, multiconditions, findings for arthritis and pain reported)	VR 45–60 min, 2–3 days/week, 6–12 weeks,	No difference compared to conventional care for knee osteoarthritis and neck pain; inconclusive for low back pain
Guo et al. [64]	Systematic review and meta-analysis	Neck pain (*n* = 8 studies, 382 subjects)	VR 20–360 min, 1–6 weeks, 1–4 weeks	Small effect pain intensity (SMD −0.51, 95% CI −0.91, −0.11); improved disability, kinesiophobia, and range of motion in VR group. High heterogeneity, limited quality; adverse event motion sickness
Ye et al. [65]	Systematic review and meta-analysis	Neck pain (*n* = 5 studies, 192 subjects)	VR	Modest improvement in pain (VAS SMD—0.58 (95% CI (−0.91—0.25) Neck Disability Index (SMD = −0.54; 95% CI (−1.24, 0.15); ROM non-significant difference
Diabetes				
Lee [71]	Randomized control trial	Diabetes (*n* = 45 subjects)	VR Three-arm study: (1) Control group: typical routine (2) IG: VR (3) IG: Exercise 40–60 min, 3x/week, 2 weeks	VR and exercise group improved mean blood glucose (F = 12.001 *p* < 0.001) and serum fructosamine (F = 3.274, *p* = 0.016) compared to the control group. Both intervention groups had significant improvement in muscle mass compared with the control group (F = 4.445, *p* = 0.003). No difference in body mass index.
Pulmonary disease				
Rutkowski et al. [72]	Randomized control trial	COPD (*n* = 106)	Three groups: (1) endurance training pulmonary rehab (ET); (2) pulmonary rehab + VR and endurance training (ET + VR); (3) pulmonary rehab + VR (VR) Endurance exercise: 20–30 min VR: 20 min Pulmonary rehab components 5 days/week X 2 weeks	ET + VR group superior to ET group in arm curl (*p* < 0.003), chair stand (*p* < 0.008), back scratch (*p* < 0.002), chair sit and reach (*p* < 0.001), up and go (*p* < 0.000), and 6-min walk test (*p* < 0.011). VR group was superior to ET group in arm curl (*p* < 0.000), chair stand (*p* < 0.001), and 6-min walk test (*p* < 0.031).
Heart Disease				
Bouraghi [68]	Systematic review	Cardiac disease (*n* = 26 studies, 1281 subjects)	VR: rehabilitation 2–3x/week, 2 weeks–6 months	Reduced pain and length of hospitalization and improved systolic blood pressure and heart rate.
García-Bravo et al. [67]	Systematic review	Cardiac rehabilitation (*n* = 10 studies, 874 subjects)	VR and Video Games + cardiac rehabilitation 20–60 min, 2–7 sessions/week, 6 weeks–12 months	Increased heart rate, less pain, a greater ability to walk, higher energy levels, an increase in physical activity, and improvements of motivation and adherence
Blasco-Peris et al. [66]	Systematic review and meta-analysis	Cardiovascular disease in cardiac rehabilitation (*n* = 8 studies, 733 subjects)	Exergaming component to cardiac rehabilitation compared to traditional cardiac rehabilitation 20–60 min, 2 sessions/day–7 sessions/week, 6–48 weeks	Non-significant statistical difference between exergaming and conventional cardiac rehab programs in exercise capacity changes (measured as the distance covered in the 6MWT).

MD: mean difference; SMD: standardized mean difference; VAS: Visual Analogue Scale; 6MWT: 6-min walk test; ADLs: activities of daily living.

## Data Availability

Data included in table and text of manuscript.

## Supplementary Materials

The following supporting information can be downloaded at https://www.mdpi.com/article/10.3390/healthcare11202751/s1: Table S1: Technology Specifications for Exoskeletons; Table S2: Technology Specifications for Augmented and Virtual Reality; Table S3: Technology Specifications for Remote Monitoring.
