# Supplementary material for: Rehabilitation Technologies for Chronic Conditions: Will We Sink or Swim?
Source: Healthcare (Basel). 2023 Oct 17;11(20):2751. doi: 10.3390/healthcare11202751 (PMC10606667; doi:10.3390/healthcare11202751)
Supplement: Supplementary file 1 [file healthcare-11-02751-s001.zip › healthcare-2589102-supplementary.pdf]

Table S1. Technology Specifications for Exoskeletons.

Author	Study Design	Use	Joints	Powered vs passive, pseudo passive, hybrid	Portable vs stationary	Materials (hard vs soft)	Degree of freedom (DOF) specified
Calafiore[43]	Systematic Review	Gait training	NS	powered and hybrid	both	hard	NS
Hsu[7]	Systematic Review + meta	Gait training	hip, knee	powered	portable	NS	NS
Karunakaran[6]	Systematic Review	Gait training	hip, knee, ankle	powered	portable	hard	NS
Leow[8]	Systematic Review	Gait training	hip, knee, ankle	powered	portable	NS	NS
Carmignano[44]	Systematic Review	Gait training	hip, knee, ankle	powered and hybrid	both	hard	NS
Picelli[45]	Systematic Review	Gait training	Lower extremity	powered	both	NS	NS
Bowman[46]	Systematic Review	Gait training	NS	powered	stationary	hard	NS
Calabro[47]	Systematic Review	Gait training	hip, knee, ankle	powered	both	hard	NS
Hayes[49]	Systematic Review	Gait training	lower extremity	powered	both	hard	NS
Zhang[50]	Systematic Review	Gait training	hip, knee, ankle	powered and non-powered	portable	hard	2*
Dijkers[48]	Review of Systematic Reviews	Gait training	NS	powered	both	hard	NS
McGibbon[51]	Randomized controlled trial	Assisted physical performance	knee	powered	portable	hard	NS

NS: Not Specified in article
*Only reported for 1 study in the review

Table S2. Technology Specifications for Augmented and Virtual Reality.

Author	Study Design	AR /VR	Immersion Type (non, fully, semi)	Tracking System	Gaming Component Present in intervention
Chu[58]	Systematic Review + Meta	VR	fully, semi, and non	fixed screens, headsets, large screen projections	both
Demeco[11]	Systematic Review	VR	fully	headsets and large screen projection	both
Gil[70]	Systematic Review + Meta	AR	non	headsets, handheld (tablets), projections	both
Heffernan[59]	Systematic Review	VR and AR	fully	headsets, large screen projector, glasses for AR	NS

Kwon[12]	Systematic Review + Meta	VR	non and semi	NS	yes
Mohamed Hazza[60]	Systematic Review + Meta	VR	fully, semi, and non	headsets, fixed screens	both
Sevcenko[10]	Systematic Review	VR	semi and non	fixed screen	yes
Blasco[61]	Systematic Review	VR	non	fixed screen	yes
Choi[62]	Systematic Review +Meta	VR	non and fully	headsets	both
Youssef[63]	Systematic Review	VR	non and fully	one fixed screen, rest unspecified	both
Guo[64]	Systematic Review +Meta	VR	NS	one used a headset, the rest unspecified	both
Ye[65]	Systematic Review +Meta	VR	non, fully, semi	head mounted devices, fixed screen	both
Lee.[71]	Randomized Controlled Trial	VR	non	fixed	no
Bouraghi [68]	Systematic Review	VR	NS	NS	NS
García-Bravo[67]	Systematic Review	VR	semi	fixed, handheld	yes
Blasco-Peris[66]	Systematic Review + Meta	VR	fully and semi	fixed, handheld, headset	both
Rutkowski [72]	Randomized Controlled Trial	VR	non and semi	fixed	yes

AR: Augmented Reality; VR: Virtual Reality; non: non immersive; fully: fully immersive; semi: semi-immersive; NS: Not Specified in article

Table S3. Technology Specifications for Remote Monitoring.

**Author**	**Study Design**	**What is monitored**	**Mode of monitoring**	**Type of sensor**
Dorsch[77]	Randomized Controlled Trial	walking activity	wearable external sensor	accelerometer
Lynch[78]	Systematic Review	physical activity	wearable external sensors, smartphone applications	accelerometers, GPS, gyroscopes
Ozden[79]	Systematic review + Meta	physical activity and exercise prescriptions	wearable external sensors, smartphone applications	NS
Christiansen[83]	Randomized Controlled Trial	physical activity	wearable external sensor	actigraph
Mehta[84]	Randomized Controlled Trial	physical activity, goals, pain	wearable external sensor, messaging system	accelerometer
Nick[91]	Systematic Review + Meta	physiological, weight, symptoms	Phone/tablet applications connected to external sensors	NS

Zhong[89]	Systematic review + Meta	exercise, physiological signs, risk factors	phone applications, wearable external sensors, and social media management platforms.	NS
Ramachandran [90]	Systematic review + Meta	exercise, physiological signs	phone applications, external wearable sensors, external non wearable sensors	pedometers, accelerometers, heart rate monitor
Maddison [92]	Randomized Controlled Trial	exercise monitoring, physiological signs, ECG, geo positional data	external wearable sensor	accelerometer, ECG
Zanaboni[87]	Randomized Controlled Trial	Exercise, self-management	external wearable sensor, website	oximetry sensor
Benzo[86]	Randomized Controlled Trial	activity	external sensors paired with tablet	actigraph sensor
Stamenova[88]	Randomized Controlled Trial	Physiological signs	Bluetooth devices: external sensors paired with a tablet	piezoelectric sensor, pulse oximetry sensor, temperature sensor
Michaud [85]	Systematic review + Meta	Physiological signs, physical activity	Phone application, external sensors (wearable and unwearable), telehealth software	NS

NS: Not Specified in article; GPS: Global Position Systems; ECG: electrocardiogram
